# Prognostic value of the expression of chemokines and their receptors in regional lymph nodes of melanoma patients

**DOI:** 10.1111/jcmm.15015

**Published:** 2020-01-26

**Authors:** Ting‐feng Xiong, Fu‐qiang Pan, Qian Liang, Ruijin Luo, Dong Li, Haiyan Mo, Xiang Zhou

**Affiliations:** ^1^ Department of Medical Treatment Cosmetology The Second Affiliated Hospital of Guangxi Medical University Nanning China; ^2^ Medical Department The Second Affiliated Hospital of Guangxi Medical University Nanning China

**Keywords:** CC, chemokine receptors, chemokines, CX3C, CXC, tumour immunity, XC

## Abstract

Chemokines and their receptors have been reported to drive immune cells into tumours or to be directly involved in the promotion or inhibition of the development of tumours. However, their expression in regional lymph node (LN) tissues in melanoma patients remains unknown. The present study investigated the relationship between the expression of mRNA of chemokines and their receptors and clinicopathology of the regional LN tissues of skin cutaneous melanoma (SKCM) patients available in The Cancer Genome Atlas. The relationship between chemokines and their receptors and the composition of immune cells within the tumour was analysed. In SKCM regional LN tissues, the high expression of 32 types of chemokines and receptors, namely CCL2, 4‐5, 7‐8, 13, 22‐25, CCR1‐9, CXCL9‐13, 16, CXCR3, 5, 6, XCL1‐2 and XCR1 in LN was associated with favourable patient prognosis. Conversely, high expression of CXCL17 was an indicator of poor prognosis. The expression of mRNA for CXCL9‐11, 13, CXCR3, 6, CCL2, 4, 5, 7, 8, 25, CCR1, 2, 5, and XCL1, 2 in regional LN tissues was positively correlated with the fraction of CD8‐positive T cells and M1 macrophages, and was negatively correlated with M0 macrophages. CCR4, 6‐9, CCL13, 22, 23 and XCR1 were positively correlated with the fraction of memory B cells and naive T cells, and negatively correlated with M0 macrophages and resting mast cells, suggesting that chemokines and their receptors may affect the prognosis of patients by guiding immune cells into the tumour microenvironment to eliminate tumour cells.

## 1 INTRODUCTION

In many malignancies, including skin cutaneous melanoma (SKCM), enhanced infiltration of the tumour by an immune cell is typically associated with good prognosis.^1^, ^2^ Tumour‐infiltrating lymphocytes (TIL) represent the response of the host organism to the tumour. When a tumour develops, the body can react by mobilizing the immune system, and the prognosis of the patient depends on whether the immune cells can generate an effective anti‐tumour response. The destruction of the tumour is dependent on the ability of immune cells to migrate to the site of its location and infiltration of the cancerous tissue. Tumour microenvironment (TME) comprises diverse cells types, including cancer stromal cells, fibroblasts, lymphocytes, granulocytes, macrophages, mast cells (MCs), natural killer cells (NKs), dendritic cells (DCs) and myeloid‐derived suppressor cells (MDSCs).^3^ In most cases, the presence of B lymphocytes, cytotoxic CD8‐positive T lymphocytes, NKs, ‘M1‐like’ macrophages and high numbers of DCs are indicative of a favourable outcome. CD8‐positive T cells are the main effector of anti‐tumour immunity. They recognize and destruct tumour cells carrying specific antigens, which are the product of the expression of mutated genes.^4^, ^5^ Conversely, ‘M2‐like’ macrophages, granulocytes, MCs, MDSCs, immature DCs, regulatory T cells (Tregs) and TH17 lymphocyte high density are associated with poor prognosis.^6^

Lymph nodes (LNs) are an integral part of the immune system in humans and are essential for the maintenance of effective immune responses. LNs are penetrated by networks of fibres formed by fibroblastic reticular cells (FRCs). These structures provide a basis for the transport of small molecules such as chemokines and soluble antigens with molecular mass less than 70 kD, enabling the mediation of inflammatory response or immune function by chemokines.^7^ Chemokines, the largest family of cytokines, constitute a class of low molecular weight secreted proteins capable of inducing directional migration of cells. When immune cells and tissue cells, including fibroblasts, endothelial cells and epidermal cells are induced by stimuli such as growth factors, interferons, and viral and bacterial products, different chemokines can be secreted.^8^, ^9^ In the TME, both tumour and immune cells express chemokines, which can lead to the spread of tumour cells. On the other hand, chemokines can promote the entry of specific immune cells into tumours, facilitating the anti‐tumour response and improving the prognosis of patients.^1^, ^10^ In this regard, the outcome of the disease in SKCM patients has been demonstrated to depend on the infiltration of lymphocytes into the tumour in SKCM patients, a process that is affected by chemokine or cytokine gradients.^11^, ^12^ The generation of an effective anti‐tumour immune response depends on the synergy between different immune cells, and their transport and distribution are co‐ordinated by the interaction between chemokines and their receptors. For example, CCL19 and CCL21 chemokines activate naive T cells, B cells, mature DC cells and NK cells via the CCR7 receptor, inducing their migration to secondary lymphoid organs (SLO).^13^ Immature DCs express CXCR1, CCR1, CCR2 and CCR6 receptors, and inflammatory chemokines acting as their ligands recruit these cells to the site of inflammation.^14^ B cells express the chemokine receptor CXCR5, and the ligand of CXCR5 promotes the homing of B cells into LNs.^14^ CD8‐positive T cells express the chemokine receptor CXCR3, which, when bound by the chemokine ligands CXCL9 and 10, drives their migration to the tumour.^15^ Increased levels of CXCL9‐11 are associated with a higher number of CD8‐positive T cells infiltrating the tumour, decreased metastatic activity and improved survival of cancer patients.^16^

CIBERSORT, an analytical tool for estimating the relative abundance of different cell types based on RNA transcripts, allows calculating cell infiltration in tissues based on gene expression profile data. In comparison with traditional methods, CIBERSORT has the advantage of simultaneous assessment of multiple types of infiltrating cells. This approach is not affected by the expression of the same surface marker by different cell types. Moreover, samples can be easily processed and stored in a standardized manner, alleviating problems that negatively affect the quality of data collected at different times and locations.^17^, ^18^ The results obtained using CIBERSORT to calculate lymphocytic infiltration are consistent with the data generated by flow cytometry, and this methodology has been applied to the study of multiple diseases.^17^, ^18^ The basic matrix in CIBERSORT, LM22, permits the relative quantitation of 22 cell types, including T cells, naive and memory B cells, plasma cells and subpopulations of myeloid cells.^19^

The research on the role of chemokines in SKCM is sporadic. The number of studies on chemokines and chemokine receptors in metastatic regional LN tissue is limited as well. In view of the paucity of relevant information, the present study focused on the relationship between mRNA expression and clinical pathology of chemokines and their receptors in regional LN tissues of 221 patients with The Cancer Genome Atlas (TCGA) SKCM. CIBERSORT was used to analyse the infiltration of SKCM by CD8‐positive and CD4‐positive T cells, DCs, B cells, macrophages, MCs, and NK cells and establish the relationship between chemokines and their receptors in the LN tissues of SKCM patients and the fraction of the immune cells in the tumour.

## 2 MATERIALS AND METHODS

### 2.1 Data acquisition

The SKCM gene expression data set available on the TCGA website (https://cancergenome.nih.gov) was downloaded from the University of California, Santa Cruz, Xena website (http://xena.ucsc.edu/). The Xena website contains TCGA ‐SKCM RNA‐seq data that are consistent with the content of the TCGA and have the additional advantage of being easier to download and organize. The data have been standardized for the RSEM (RNA‐Seq by Expectation‐Maximization) conversion. Together, 472 gene expression data sets from 470 patients were retrieved (some gene expressions data sets were taken from the same patient).

**Figure 1:**
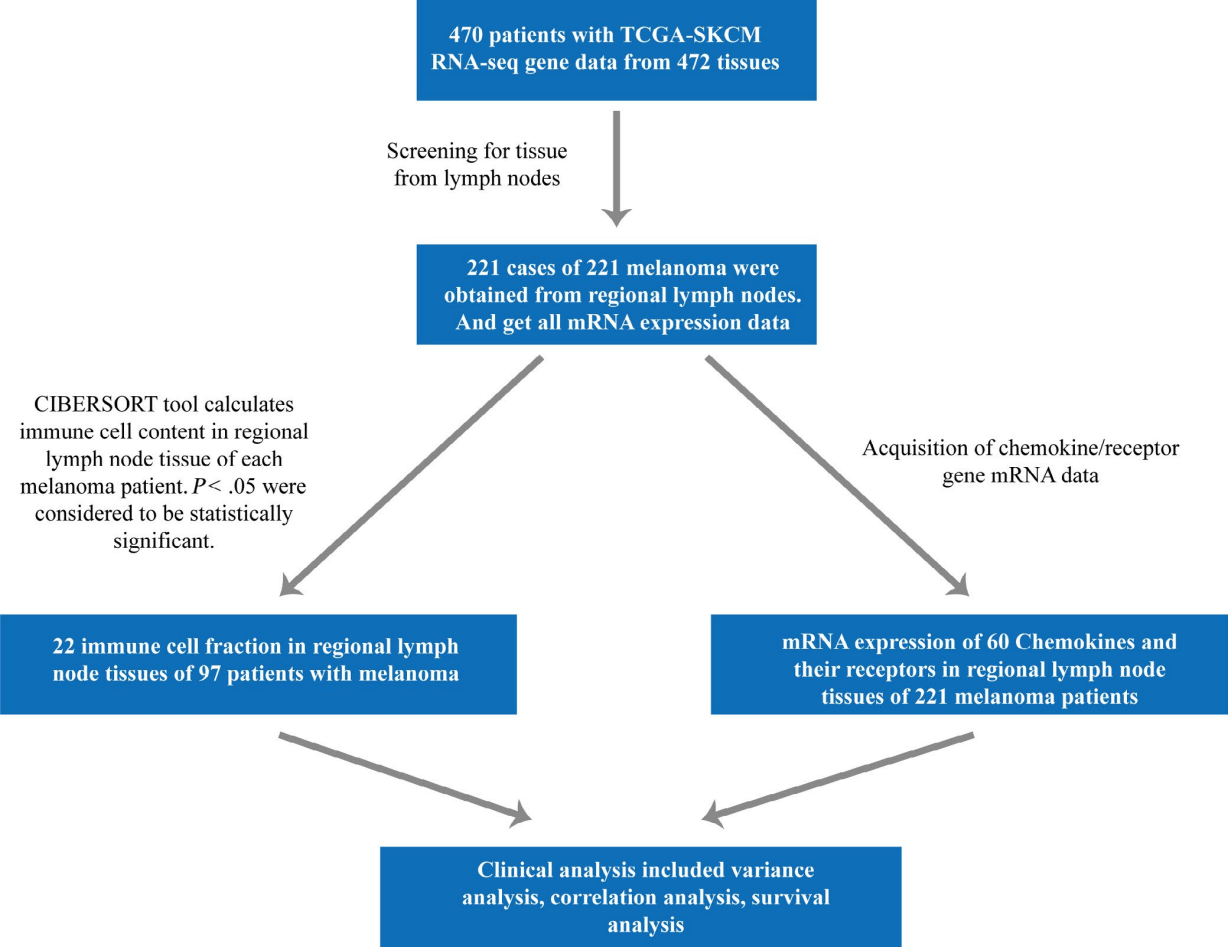
Study flowchart

The 470 cases comprised 221 cases of LN tissue of SKCM patients, 103 cases of in situ SKMC tissue, 68 cases of distant metastatic tissue, 74 cases of adjacent tissue and three cases of not identified tissue sources. Subsequently, the gene expression data of LN tissue were screened for analysis, and complete gene expression values and clinical information of 221 SKCM patients were obtained. The clinical data included height, weight, age, BIM, pathological grade, TMN staging. See Clinical Study flow chart (Figure 1) and Clinicopathological features (Table 1).

**Table 1:** Clinicopathological features of melanoma patients

Variable	n	%
Sex		
Female	83	37.56
Male	138	62.44
Age (y)		
<55	107	48.42
≥55	111	50.23
Missing	3	1.36
Pathological stage		
Stage 0/I/II	94	42.53
Stage III/IV	103	46.61
Missing	24	10.86
T stage		
T3/T4	81	36.65
Tis/T0/T1/T2/T3	103	46.61
TX/missing	37	16.74
N stage		
N0	103	46.61
N1/N2/N3	100	45.25
NX/missing	18	8.14
M stage		
M0	201	90.95
M1	8	3.62
Missing	12	5.43

Abbreviations: M, distant metastasis; N, Lymph node metastasis; T, Local invasion stage.

### 2.2 Evaluation of immune cell components using CIBERSORT

The CIBERSORT website (http://cibersort.stanford.edu) provides R language computing source code, as well as the basic matrix (LM22). The R language programs include preprocessCore and BiocManager package. The statistical rank was set to 1000 (recommended value is >100) in the R language program, and quantile normalization was disabled. Subsequently, the lymphocyte infiltration ratio of 22 distinct cell types was calculated, with the sum of fractions equal 1. *P*‐value was determined for the tissue infiltration score in each patient, and *P* < .05 were considered to be statistically significant.

### 2.3 LASSO scores for chemokine and its receptor mRNA expression

Survival analysis and univariate Cox regression were used to screen for chemokines and receptors with prognostic value, since strong multicollinearity may be present between chemokines and their receptors. Therefore, the LASSO scoring based on the selected chemokines and receptors was performed first, and subsequently, multi‐factor Cox regression coefficients were calculated to establish a risk‐scoring model.^20^

### 2.4 Statistics

To compare differences between groups calculated by survival analysis or Cox regression, continuous variables (including age, chemokine and its receptor mRNA expression value, and the LASSO score) had to be converted into two categorical variables. For the expression of chemokine and its receptor mRNA, and for the LASSO score, the ‘survminer’ package in the R language was used to calculate the best cut‐off value higher than the cut‐off value for the high expression group and lower than the cut‐off value for the low expression group.

Heatmaps were drawn using EXCEL 2016 (Microsoft Corp) and Adobe Illustrator (Adobe Inc). The R software was used to perform the screening of genetic and clinical data screening, as well as statistical calculations. Wilcoxon rank sum test was used to determine differences in clinical pathology and mRNA expression data; *P* < .01 was considered statistically significant. Correlation between chemokines and their receptors was established by Spearman rank correlation analysis using the ‘cor.test’ function in R software; *P* < .01 was considered statistically significant. For survival analyses, the Kaplan‐Meier method with log‐rank test was used and the survival curves were plotted by the R software.

## 3 RESULTS

### 3.1 Relationship between the expression of chemokines and their receptors mRNA and clinicopathological data

The expression of chemokines and their receptors mRNA in SKCM regional LN tissues did not differ significantly among the T, M, N and AJCC staging (*P* > .01; Table 2).

**Table 2:** Compare of the differences between the expression of RNA of chemokines and their receptors and clinicopathological data

Gene	T	N	M	Pathological stage	Gene	T	N	M	Pathological stage
CCL1	0.25	0.88	0.79	0.72	CCR7	0.9	0.29	0.96	0.6
CCL2	0.06	0.34	0.52	0.65	CCR8	0.68	0.17	0.36	0.41
CCL3	0.47	0.56	0.6	0.96	CCR9	0.36	0.12	0.59	0.38
CCL4	0.12	0.23	0.38	0.51	CCR10	0.33	0.05	0.28	0.05
CCL5	0.51	0.3	0.27	0.68	CXCL1	0.25	0.06	0.62	0.11
CCL7	0.04	0.43	0.49	0.45	CXCL10	0.08	0.35	0.96	0.71
CCL8	0.05	0.13	0.49	0.27	CXCL11	0.16	0.43	0.86	0.67
CCL11	0.75	0.59	0.82	0.77	CXCL12	0.67	0.43	0.27	0.4
CCL13	0.11	0.14	0.98	0.43	CXCL13	0.45	0.98	0.31	0.77
CCL14	0.5	0.95	0.36	0.88	CXCL14	0.39	0.56	0.96	0.86
CCL15	0.84	0.65	0.48	0.83	CXCL16	0.15	0.13	0.82	0.36
CCL16	0.4	0.86	0.94	0.7	CXCL17	0.76	0.43	0.85	0.52
CCL17	0.57	0.82	0.61	0.6	CXCL2	0.05	0.33	0.14	0.48
CCL18	0.86	0.07	0.49	0.35	CXCL3	0.48	0.74	0.16	0.91
CCL19	0.62	0.55	0.95	0.97	CXCL5	0.52	0.6	0.26	0.71
CCL20	0.48	0.28	0.97	0.51	CXCL6	0.48	0.8	0.79	0.93
CCL21	0.86	0.77	0.91	0.99	CXCL8	0.83	0.75	0.37	0.56
CCL22	0.33	0.43	0.82	0.86	CXCL9	0.17	0.58	0.76	0.86
CCL23	0.32	0.11	0.39	0.36	CXCR1	0.69	0.8	0.86	0.69
CCL24	0.11	0.75	0.61	0.21	CXCR2	0.69	0.66	0.91	0.82
CCL25	0.19	0.81	0.56	0.32	CXCR3	0.33	0.32	0.33	0.56
CCL26	0.98	0.26	0.27	0.45	CXCR4	0.32	0.43	0.29	0.47
CCL27	0.84	0.69	0.63	0.6	CXCR5	0.97	0.46	0.89	0.83
CCL28	0.41	0.87	0.35	0.64	CXCR6	0.16	0.27	0.41	0.46
CCR1	0.01	0.17	0.55	0.37	CXCR7	0.23	0.25	0.47	0.49
CCR2	0.42	0.27	0.21	0.44	CX3CL1	0.84	0.61	0.47	0.56
CCR3	0.72	0.23	0.88	0.34	CX3CR1	0.36	0.27	0.72	0.35
CCR4	0.96	0.4	0.43	0.58	XCL1	0.17	0.05	0.24	0.3
CCR5	0.14	0.35	0.43	0.55	XCL2	0.22	0.04	0.35	0.23
CCR6	0.88	0.84	0.26	0.97	XCR1	0.6	0.45	0.35	0.53

Note

Compare the differences between the expression of RNA of chemokines and their receptors and clinicopathological data were analysed by Wilcoxon rank test. *P* < .01 was considered statistically significant. T: Tis/T0/T1/T2 (n = 103) VS T3/T4 (n = 81), N: N0 (n = 103) VS N1/N2/N3 (n = 100), M: M0 (n = 201) VS M1 (n = 8), Pathological Stage: 0/I/II (n = 94) VS III/IV (n = 103).

Abbreviations: M, distant metastasis; N, lymph node metastasis; T, local invasion stage; VS, versus.

### 3.2 The effect of expression of chemokines and their receptors mRNA on the survival of SKCM patients

**Figure 2:**
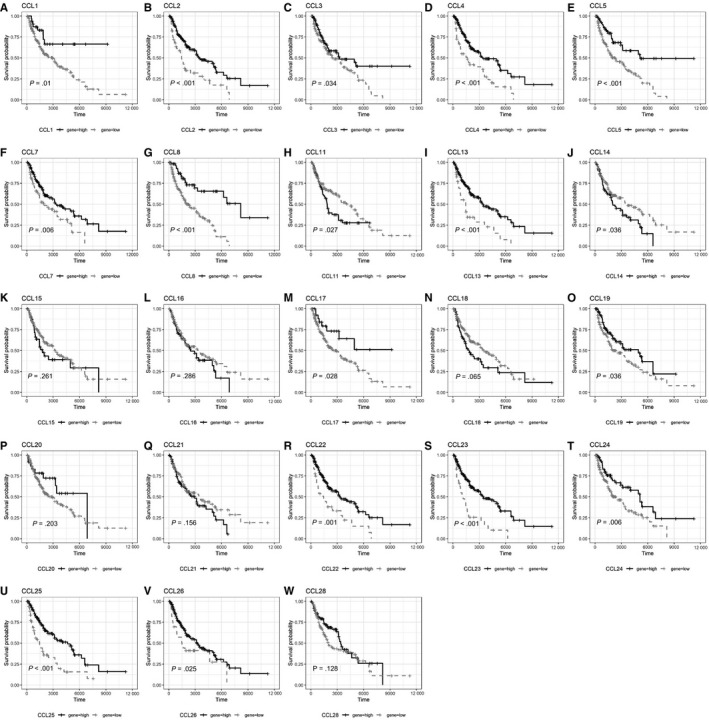
Survival curve of CCL subgroup A, CCL1, B, CCL2, C, CCL3, D, CCL4, E, CCL5, F, CCL7, G, CCL8, H, CCL11, I, CCL13, J, CCL14, K, CCL15, L, CCL16, M, CCL17, N, CCL18, O, CCL19, P, CCL20, Q, CCL21, R, CCL22, S, CCL23, T, CCL24, U, CCL25, V, CCL26, W, CCL28

**Figure 3:**
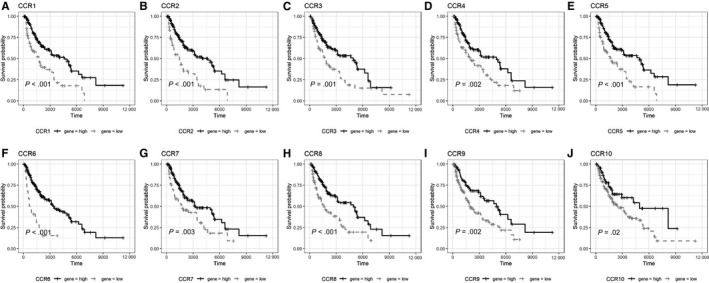
Survival curve of CCR subgroup A, CCR1, B, CCR2, C, CCR3, D, CCR4, E, CCR5, F, CCR6, G, CCR7, H, CCR8, I, CCR9, J, CCR10

**Figure 4:**
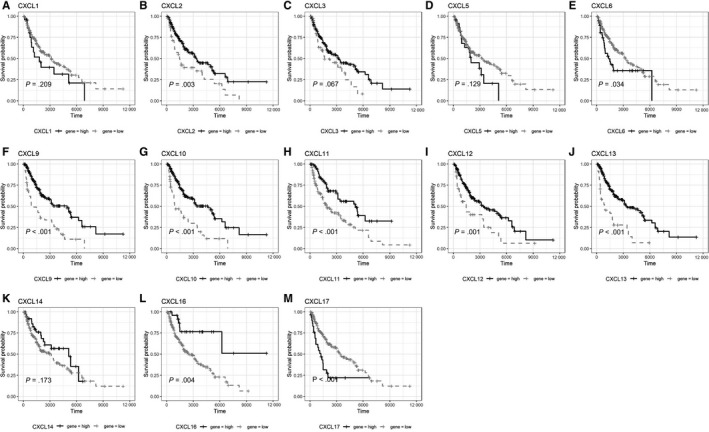
Survival curve of CXCL subgroup A, CXCL1, B, CXCL2, C, CXCL3, D, CXCL5, E, CXCL6, F, CXCL9, G, CXCL10, H, CXCL11, I, CXCL12, J, CXCL13, K, CXCL14, L, CXCL16, M, CXCL17

**Figure 5:**
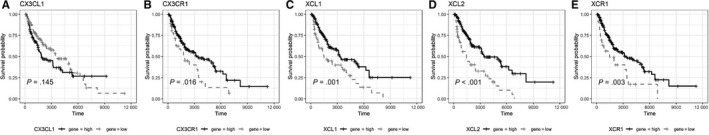
Survival curve of CXCR subgroup A, CXCR1, B, CXCR2, C, CXCR3, D, CXCR4, E, CXCR5, F, CXCR6, G, CXCR7

**Figure 6:**
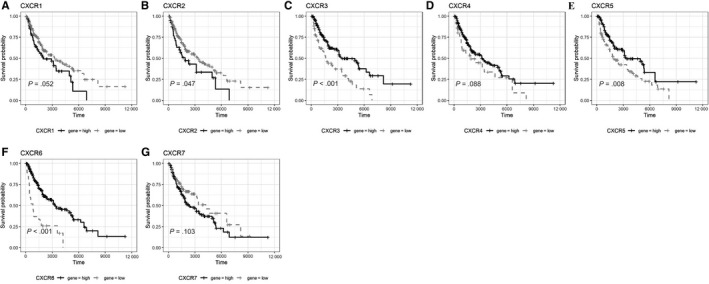
Survival curve of CX3CL/CX3CR and XCL/XCR A, CX3CL1, B, CX3CR1, C, XCL1, D, XCL2, E, CCR1

To compare the survival of SKCM patients with high and low expression of chemokines and their receptors mRNA, univariate Cox regression was performed, and survival curves were plotted (Table 3; Figures 2, 3, 4, 5 and 6). The difference in survival between the groups was statistically significant (*P* < .01). Among the 33 analysed genes, only in the case of CXCL17 the high expression group had worse survival prognosis than the low expression group. For the remaining 32 chemokine/receptor, high expression was associated with a better survival rate of the patients**.**

**Table 3:** Univariate COX risk regression analysis

Gene	Hazard ratio	95% CI	*P*‐value	Gene	Hazard ratio	95% CI	*P*‐value
CCL1	2.59	1.26‐5.32	.01	CCR6	3.55	2.11‐5.97	<.001
CCL11	0.64	0.44‐0.95	.027	CCR7	1.81	1.22‐2.68	.003
CCL13	2.35	1.53‐3.61	<.001	CCR8	2.18	1.49‐3.2	<.001
CCL14	0.66	0.45‐0.97	.036	CCR9	1.92	1.28‐2.89	.002
CCL15	0.77	0.48‐1.22	.261	CX3CL1	0.75	0.52‐1.1	.145
CCL16	0.81	0.54‐1.2	.286	CX3CR1	1.74	1.11‐2.73	.016
CCL17	2.25	1.09‐4.63	.028	CXCL1	0.7	0.4‐1.22	.209
CCL18	0.68	0.46‐1.02	.065	CXCL10	2.58	1.73‐3.86	<.001
CCL19	1.52	1.03‐2.25	.036	CXCL11	2.2	1.46‐3.32	<.001
CCL2	2.19	1.46‐3.27	<.001	CXCL12	2.03	1.33‐3.22	.001
CCL20	1.56	0.79‐3.09	.203	CXCL13	3	1.92‐4.7	<.001
CCL21	0.76	0.52‐1.11	.156	CXCL14	1.45	0.85‐2.47	.173
CCL22	2.13	1.38‐3.3	.001	CXCL16	3.35	1.47‐7.65	.004
CCL23	2.77	1.76‐4.36	<.001	CXCL17	0.41	0.25‐0.67	<.001
CCL24	1.84	1.19‐2.84	.006	CXCL2	1.85	1.23‐2.79	.003
CCL25	2.43	1.65‐3.59	<.001	CXCL3	1.53	0.97‐2.43	.067
CCL26	1.73	1.07‐2.8	.025	CXCL5	0.65	0.38‐1.13	.129
CCL28	1.36	0.91‐2.03	.128	CXCL6	0.6	0.37‐0.96	.034
CCL3	1.54	1.03‐2.28	.034	CXCL9	2.56	1.72‐3.79	<.001
CCL4	2.14	1.44‐3.16	<.001	CXCR1	0.67	0.45‐1	.052
CCL5	2.44	1.54‐3.89	<.001	CXCR2	0.63	0.41‐0.99	.047
CCL7	1.72	1.17‐2.53	.006	CXCR3	2.09	1.43‐3.07	<.001
CCL8	3.31	1.93‐5.67	<.001	CXCR4	1.49	0.94‐2.34	.088
CCR1	2.26	1.54‐3.32	<.001	CXCR5	1.67	1.14‐2.43	.008
CCR10	1.73	1.09‐2.76	.02	CXCR6	3.27	2.03‐5.28	<.001
CCR2	2.52	1.69‐3.75	<.001	CXCR7	0.7	0.46‐1.08	.103
CCR3	1.88	1.28‐2.76	.001	XCL1	1.9	1.29‐2.8	.001
CCR4	1.81	1.24‐2.65	.002	XCL2	2.45	1.67‐3.61	<.001
CCR5	2.24	1.53‐3.29	<.001	XCR1	2	1.26‐3.18	.003

Note

*P* < .01 was considered statistically significant.

Abbreviation: 95% CI, 95% confidence interval.

### 3.3 The relationship between LASSO scores for the expression of mRNA of chemokines and their receptors and the survival of SKCM patients

**Figure 7:**
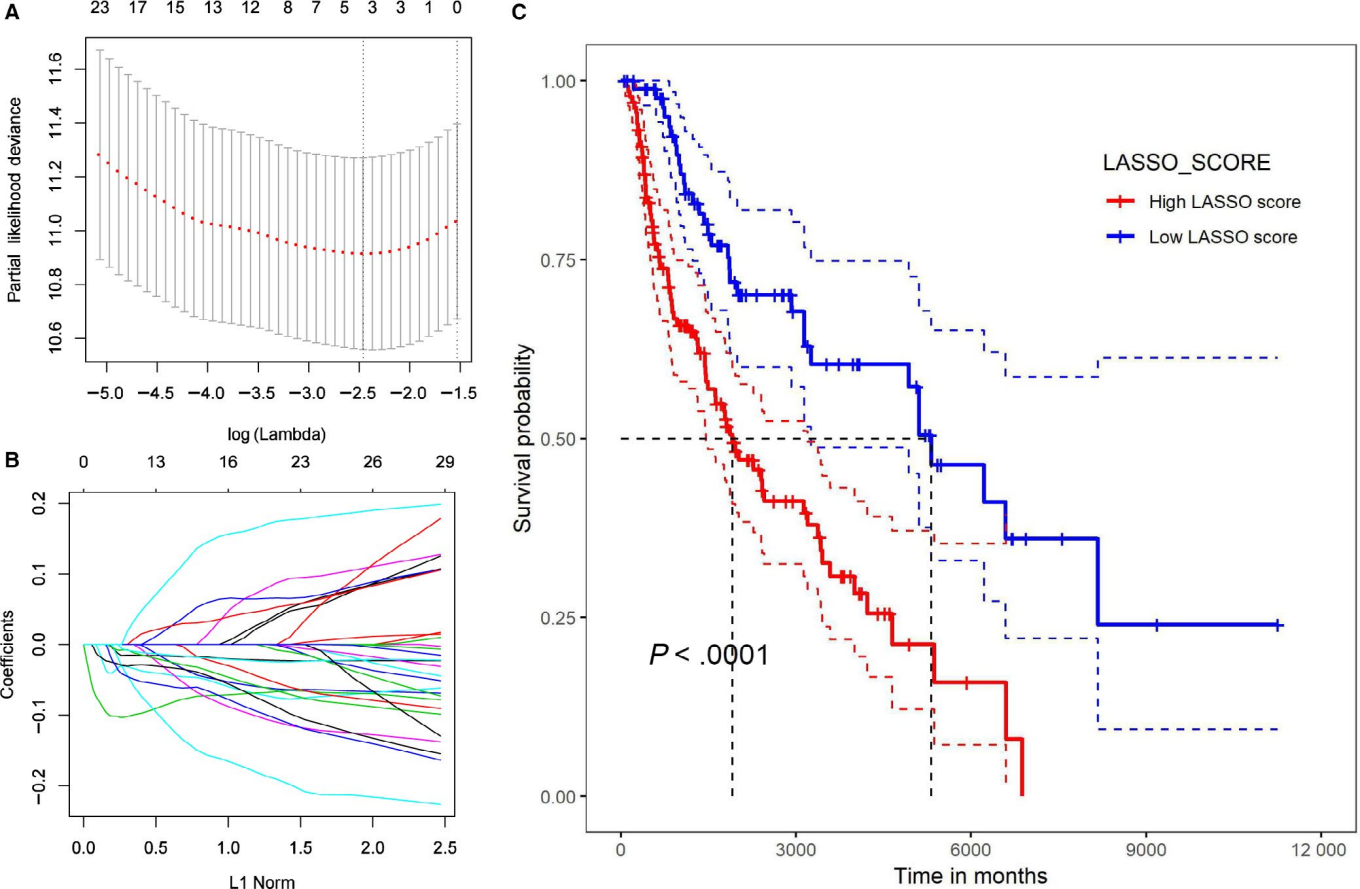
LASSO score A, Results of the LASSO model after 10‐fold cross‐validation. B, Distribution of LASSO coefficients for 22 types of immune cells. C, Survival curves of patients with high and low LASSO scores

Univariate screening of the 32 chemokine/receptor (CCL2, 4‐5, 7‐8, CCL22‐25, CCR1‐9, CXCL2‐3, 5, 9‐13, 16, XCL1‐2, and XCR1), for which higher expression was associated with a better prognosis. As there was a significant correlation between these 32 genes (Tables S1‐S3, Figure S1), multicollinearity between chemokines/receptors leads to bias in multivariate COX analysis. Therefore, LASSO score was calculated before multivariate COX analysis. A total of four chemokine/receptor gene pairs were included in the LASSO score: CCL8, CCL2, CXCL10 and CCL16 (Figure 7A,B). The formula obtained was as follows: LASSO score = −(0.097 * CCL8 expression) − (0.038 * CCL2 expression) − (0.023 * CXCL10 expression) − (0.001 * CCL16 expression). The mean value of the Lasso score was −1.36 ± 0.47 (range: −2.46 to 0.43). And those four of the chemokines were negatively correlated with the LASSO score.

Univariate survival analysis was then performed with respect to the LASSO score. The cut‐off value of the LASSO score (−1.080674) was calculated using the R software and was used to divide the patient groups into a high LASSO score group and a low LASSO score group. Survival curves were plotted and indicated that the median survival time in the high LASSO score group was 1460, and 5107 days in the low LASSO score group (*P* < .001 by the log‐rank test; Figure 7C).

### 3.4 Relationship between the chemokine/receptor and infiltration of tumour by immune cells

**Figure 8:**
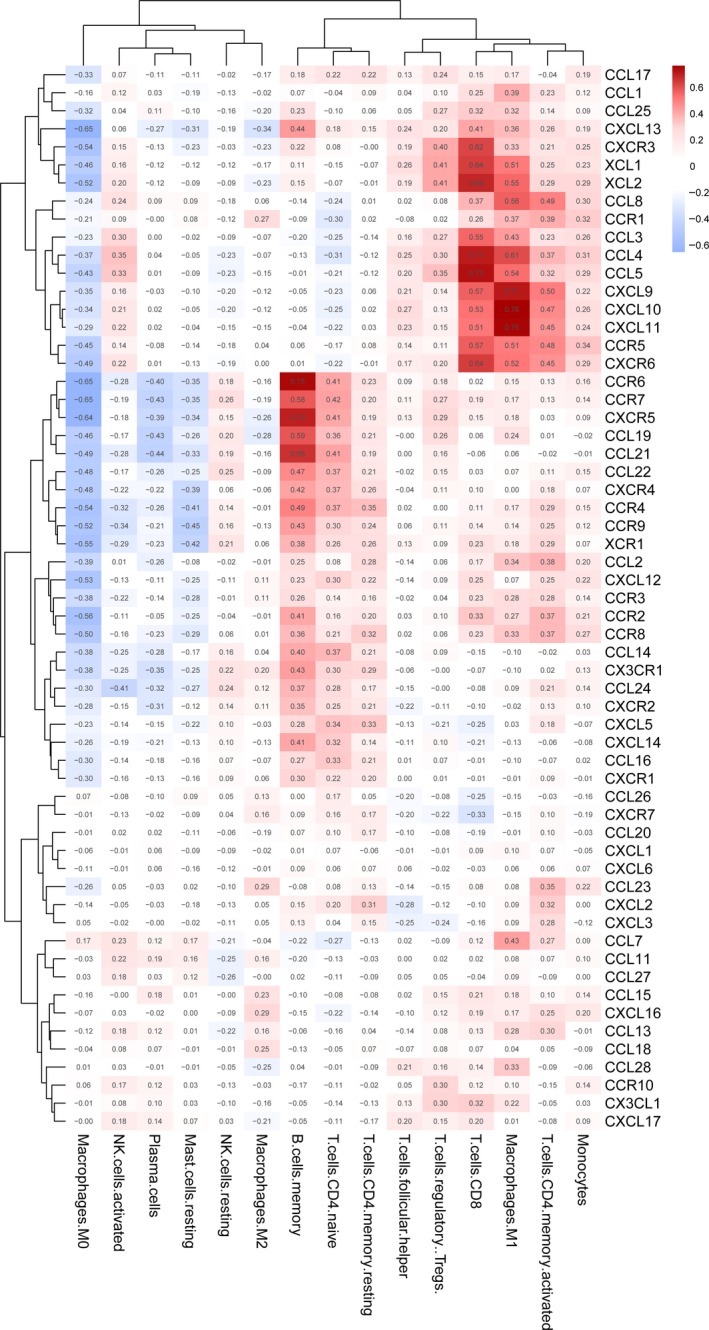
Relationship between chemokines and their receptors and tumour‐infiltrating immune cells

The CIBERSORT algorithm was utilized to calculate the infiltration scores of 22 types of immune cells in LN tissue of SKCM patients. We then calculated the relationship between chemokine/receptor and tumour immune cell fraction (Figure 8; Table S3).

### 3.5 Multivariate survival analysis

Univariate COX regression analysis showed that the survival of SKCM patients differed in a statistically significant manner with age T, N, CXCL17 and LASSO scores (*P* < .01). However, the patients with the M1 stage pathology were too small to evaluate the statistical significance of potential differences. Multivariate analysis showed that T stage, N stage, CXCL17 and LASSO scores had independent prognostic value (*P* < .01) (Table 4).

**Table 4:** Multivariate Cox regression analysis

Factors	Univariate analysis			Multivariate analysis		
	HR	95% CI	*P* value	HR	95% CI	*P* value
Sex	1.19	0.79‐1.79	.406			
Age	1.99	1.34‐2.96	.001	1.68	1.06‐2.67	.027
T	0.45	0.3‐0.69	<.001	0.53	0.33‐0.84	.007
N	2.15	1.42‐3.26	<.001	1.9	1.45‐8.15	<.001
M	1.53	0.48‐4.85	.471			
CXCL17	0.41	0.25‐0.67	<.001	0.4	0.22‐0.73	.003
LASSO score	0.39	0.25‐0.6	<.001	0.39	0.24‐0.65	<.001

## 4 DISCUSSION

Chemokines, the key mediators of the immune response, are essential for the recruitment of many different types of cells to TME.^9^ To identify their functions in SCKM, we first was assessed between the survival of the patients and the expression of chemokines and their receptors genes, using the data for SCKM regional LN tissue available in the public TCGA database. The survival prognosis of the patients with high expression of 32 chemokines and receptors (CCL2, 4‐5, 7‐8, CCL22‐25; CCR1‐9; CXCL2‐3, 5, 9‐13, 16; XCL1‐2, XCR1) was found to be better than in the low expression group. Subsequently, a multivariate COX analysis was performed since the strong correlation among these 32 chemokines/receptors might have compromised the validity of the multivariate COX regression analysis due to multiple linearity. Additionally, LASSO regression based on these 32 chemokines was computed, and analysis was performed using a comprehensive LASSO score. This approach has shown that the LASSO score was an independent prognostic factor. Also, this analysis explained the basis for the absence of a significant difference in chemokine/receptor classification in different pathological stages, suggesting that these 32 chemokine/receptor genes have independent survival prognostic significance.

Among the chemokines and receptors analysed, we found CCR4, 6‐9, CCL13, ‐22, ‐23, and XCR1 were positively correlated with the fractions of memory B cells and the naive T cells, and negatively correlated with M0 type macrophages and resting type MCs. A study on the correlation between the mature B cells in the tumour and the prognosis of malignant SKCM documented that that humoral immunity participates in the anti‐tumour defence.^21^, ^22^ Immunohistochemistry of human SKCM samples demonstrated that most tumour tissues contain a large number of infiltrating CD20‐positive cells, which are considered to be B lymphocytes, which are dispersed in the matrix surrounding the tumour.^22^ The percentage of B cell infiltration in and around the tumour was also positively correlated with patient survival.^22^, ^23^ Naive T cells express CCR7 and control the migration of immune cells to SLO. This process is mediated by the CCL19 and CCL21 receptors. Thus, CCR7 plays an important role in lymphocyte homing to LNs and spleen.^24^ It has been demonstrated that in CCR7‐deficient mice T lymphocytes are absent in the lymphoid white pulp of LNs and spleen, and are present only in the red pulp of the spleen.^25^, ^26^ Tumour‐associated MCs can release a variety of cytokines, chemokines and growth factors, promoting tumour development by enhancing angiogenesis and remodelling tumour extracellular matrix.^27^, ^28^, ^29^

The current study demonstrated also that CXCL9‐11, CXCR3, 6, CCL2, 4, 5, 7, 8, 25, CCR1, 2, 5, and XCL1, 2 are positively correlated with the fractions of M1 macrophages and CD8‐positive T cells, and negatively correlated with M0 macrophages. CD8‐positive T cells recognize tumour cell antigens and drive anti‐tumour response by secreting effector cytokines, releasing cytotoxic molecules such as granzyme B and perforin, and inducing apoptosis in tumour cells.^30^ The CXCL9‐11/CXCR3 axis regulates CD8‐positive T cell migration, differentiation and activation.^16^, ^31^ CD8‐positive T cells express CXCR3 and are capable of invading into tumours when activated by chemokines. Elevated levels of CXCL9 and CXCL10 are associated with an increased number of tumour‐infiltrating CD8‐positive T cells, decreased cancer metastasis and increased survival of cancer patients.^32^, ^33^ Experiments on CXCR3 knockout mice bearing B16 melanoma demonstrated a critical role for CXCR3 in the migration of CD8‐positive cells. These transgenic mice exhibited significant tumour growth and decreased survival.^34^ Analysis of CD8‐positive T cells early in tumour development has a better prognostic value than the traditional staging. Most patients with stage I and stage II cancer lack T cell infiltration and are prone to disease recurrence within 5 years. T cell infiltration of the tumour is correlated with longer disease‐free survival in stage III cancer patients.^35^ In addition, CXCR3 and its ligands CXCL9 and CXCL10 are closely related to the TH1 immune response, and CXCR3 mediates the anti‐tumour response by recruiting into tumours NK cells, CD4‐positive Th1 cells and CD8‐positive cytotoxic T lymphocytes tumours. M1 macrophages express and secrete pro‐inflammatory peptides, chemokines and other effector molecules, including IL‐1, IL‐6, TNF, IL‐23 and i‐NOS, contributing to the development of anti‐tumour response.^36^ The chemokine CCL2 is a major player in this process, and its binding to the CCR2 receptor directly mediates monocyte recruitment to inflammation site and primary tumour tissue.^30^ In addition, a prolonged survival was observed in metastatic SKCM patients with high expression of CCL4, CCL5, CXCL9, CXCL10 and CXCL11^37^ and related to patients with higher response to ipilimumab treatment.^38^ The CCR5‐deficient mice use of CCR5 blockers was associated with a decrease in Treg cells, which have a tumour‐promoting effect.^39^

CXCL17 is highly expressed in a variety of cancer cells, recruiting MDSCs into tumours and partially promoting tumour growth by enhancing angiogenesis.^40^ These findings are consistent with the shorter survival of SKCM patients with high CXCL17 documented in the current work.

In conclusion, we have demonstrated that high expression of 32 chemokines and receptors (CCL2, 4‐5, 7‐8, CCL22‐25, CCR1‐9, CXCL2‐3, 5, 9‐13, 16, XCL1‐2 and XCR1) in SKCM regional LN tissue is associated with a good prognosis, which may be related to the attraction of immune cells to the TME and elimination of tumour cells. Conversely, high expression of CXCL17 is indicative of a poor prognosis.

Some limitations of the study should be acknowledged. The validity of the conclusions reached should be confirmed by an investigation involving a larger number of cases. Further analysis is also needed for each subgroup; this was not performed due to a limited number of cases in each subgroup. Other potential factors, such as the size of the tumour and the impact of anti‐cancer drugs on chemokines, need further consideration, as does the interaction between chemokines and their receptors and other signalling pathways. Finally, due to the retrospective nature of this study, the choice of offset cannot be avoided.

Immunotherapy is currently used to treat cancer, but this type of therapy is effective only for specific populations of patients.^41^, ^42^, ^43^, ^44^ Novel, more powerful treatments are urgently needed. Understanding the role of chemokines in tumour resistance to immunologic defences of the body is essential for the development of new targeted therapeutics in the future.

## CONFLICT OF INTEREST

There are no conflicts of interest.

## AUTHOR CONTRIBUTIONS

ZX and PF.Q designed the experiments; LQ and MH.Y analysed the data; XT.F, PF.Q and ZX wrote the paper. All other authors participated in revising the paper and finalizing the paper. All authors read and approved the final manuscript.

## Data Availability

All data sets generated/analysed for this study are included in the manuscript and the Supplementary Files.
