# Supplementary figures and images for: Prognostic value of the expression of chemokines and their receptors in regional lymph nodes of melanoma patients
Source: J Cell Mol Med. 2020 Jan 26;24(6):3407–18. doi: 10.1111/jcmm.15015 (PMC7131952; doi:10.1111/jcmm.15015)

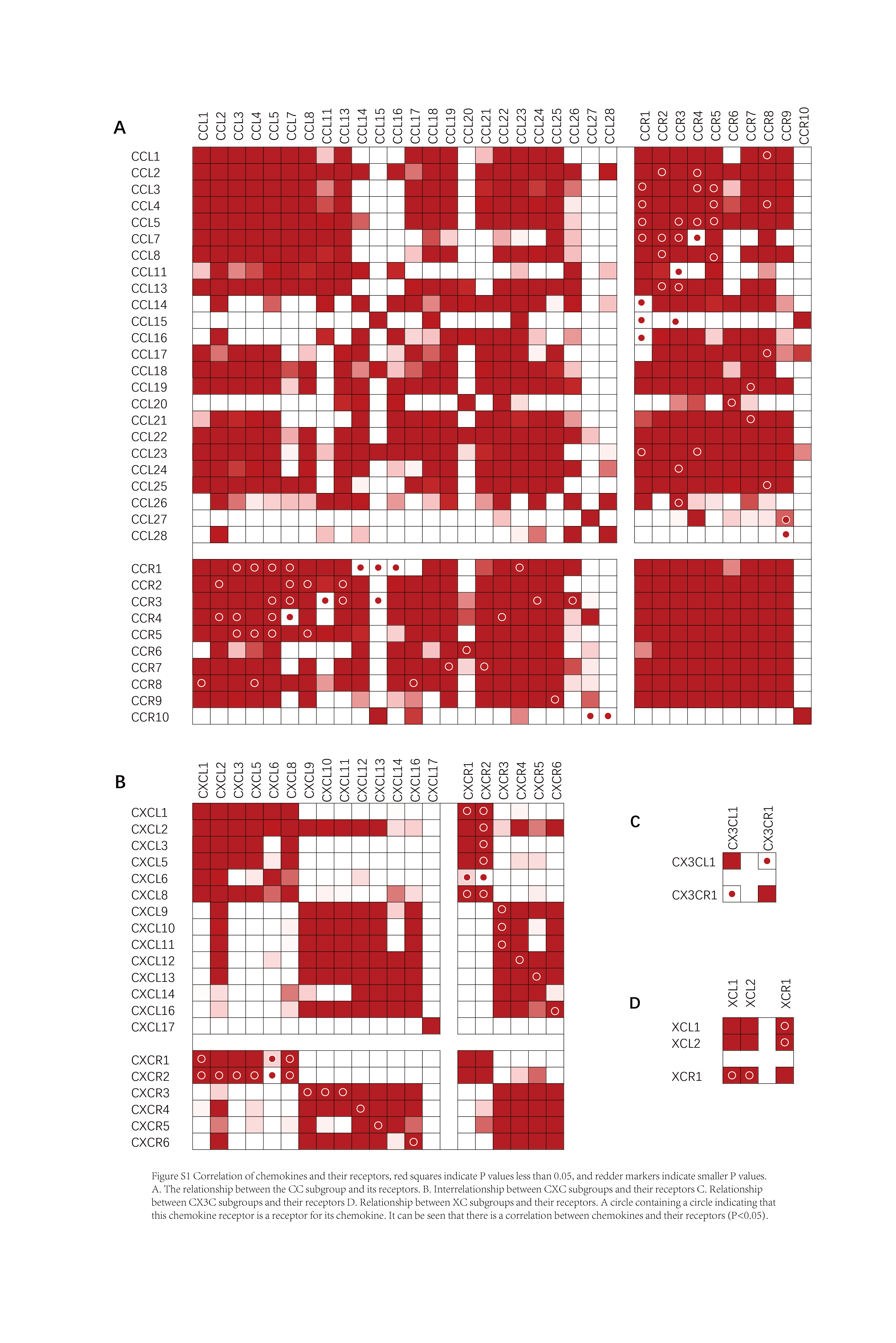

Supplement: Supplementary file 1 [file JCMM-24-3407-s001.tif]
